# Personalized functional profiling using *ex-vivo* patient-derived spheroids points out the potential of an antiangiogenic treatment in a patient with a metastatic lung atypical carcinoid

**DOI:** 10.1080/15384047.2021.2021042

**Published:** 2022-02-22

**Authors:** Hichul Kim, Victoria El-Khoury, Nadine Schulte, Tianzuo Zhan, Johannes Betge, Loic Cousin, Emanuele Felli, Patrick Pessaux, Arnaud Ogier, Oliver G Opitz, Bosung Ku, Matthias P Ebert, Yong-Jun Kwon

**Affiliations:** a Early Discovery and Technology Development, Ksilink, Strasbourg, France; b Personalized Therapy Discovery, Department of Oncology, Luxembourg Institute of Health, Dudelange, Luxembourg; c Luxembourg Center of Neuropathology (LCNP), Department of Oncology, Luxembourg Institute of Health, Dudelange, Luxembourg; d Department of Medicine II, University Medical Center Mannheim, Medical Faculty Mannheim, Heidelberg University, Mannheim, Germany; e Junior Clinical Cooperation Unit Translational Gastrointestinal Oncology and Preclinical Models, German Cancer Research Center (DKFZ), Heidelberg, Germany; f Department of Visceral and Digestive Surgery, Nouvel Hopital Civil, University Hospital of Strasbourg, Strasbourg, France; g IHU-Strasbourg, Institute of Image-guided Surgery, Strasbourg, France; h Inserm Institute of Viral and Liver Disease (INSERM U1110), Strasbourg, France; i Coordinating Unit for Digital Medicine Baden-Württemberg (KTBW), Medical Faculty Mannheim, Heidelberg University, Mannheim, Germany; j Central R&D Center, Medical & Bio Decision (MBD), Suwon, Republic of Korea

**Keywords:** Personalized functional profiling, drug screening, pharmacotyping, personalized medicine, precision medicine, spheroids, neuroendocrine tumors, lung carcinoid, antiangiogenic therapy

## Abstract

Lung carcinoids are neuroendocrine tumors representing 1 to 2% of lung cancers. This study outlines the case of a patient with a metastatic lung atypical carcinoid who presented with a pleural effusion and progression of liver metastases after developing resistance to conventional treatments. Personalized functional profiling (PFP), i.e. drug screening, was performed in *ex-vivo* spheroids obtained from the patient’s liver metastasis to identify potential therapeutic options. The drug screening results revealed cediranib, an antiangiogenic drug, as a hit drug for this patient, from a library of 66 Food and Drug Administration (FDA)-approved and investigational drugs. Based on the PFP results and the reported evidence of clinical efficacy of bevacizumab and capecitabine combination in gastro-intestinal neuroendocrine tumors, this combination was given to the patient. Four months later, the pleural effusion and pleura carcinosis regressed and the liver metastasis did not progress. The patient experienced 2 years of a stable disease under the PFP-guided personalized treatment.

## Introduction

Lung neuroendocrine tumors represent approximately 20% of all lung cancers. They are comprised of four subtypes: typical carcinoids, atypical carcinoids, large‐cell neuroendocrine carcinomas and small cell lung carcinomas.^1^ One to two percent of lung cancers is carcinoids.^2^ The incidence of lung and gastroenteropancreatic (GEP) neuroendocrine tumors (NET) has significantly risen over the last 40 years, likely due to improved diagnosis.^3^

The treatment of GEP-NET has achieved considerable advances in the last decades with the introduction of sunitinib, everolimus, somatostatin analogs and peptide receptor radionuclide therapy (PRRT) (for somatostatin receptor-positive GEP-NET) in the therapeutic scheme.^3,4^ However, everolimus is still the only treatment approved by the US Food and Drug Administration (FDA) for patients with lung NET, in particular those suffering from advanced, progressive, nonfunctional pulmonary NET, highlighting the need for more treatment options in this indication.^1,3,5,6^

One of the major challenges in managing cancer in general and lung NET in particular is identifying personalized treatment strategies that increase patients’ chances to benefit from anticancer therapy. The scarcity of this type of lung tumor calls for the contribution of multidisciplinary experts in the management of the disease.^2^ The treatment of a metastatic lung carcinoid tumor is not expected to be curative but aims at relieving the symptoms caused by tumor growth or hormonal production.^2^ So far, clinical trials tackling the management of advanced stage pulmonary carcinoids remained limited,^7^ and personalized drug screens on patient’s tumor material, i.e. functional tumor profiling or pharmacotyping^8,9^ are thus highly encouraged to issue treatment recommendations. Here we report the case of a patient with a metastatic lung NET who underwent personalized functional profiling of his tumor and was treated based on the drug screening results.

## Case presentation

A 52-year-old man was diagnosed with an atypical carcinoid of the lung (pT2 pN1 (1/25) G2, 10 mitoses/10 high power fields (HPF), Ki-67 = 15%) in June 2009. A right lower bilobectomy and systematic lymphadenectomy were then performed. In May 2012, the patient was admitted to Mannheim University Medical Center to undergo a biopsy of new lesions found in surveillance imaging and suspicious of disseminated osteoplastic bone metastases. The immunohistochemical analysis of bone material showed strong and continuous expression of chromogranin A and weak but specifically membrane-bound co-expression of CD56. The tumor cells were negative for cytokeratin 7 (CK-7), cytokeratin 20 (CK-20), thyroid transcription factor 1 (TTF-1), napsin A, prostate specific antigen (PSA), and prostate specific acid phosphatase (PSAP) staining. The mitotic count was 4 per 10 HPF and the Ki-67 index ranged between 10% and 15%. The diagnosis of a disseminated hepatic and bone metastasis due to the clinically known lung atypical carcinoid was confirmed. The patient was treated with capecitabine and temozolomide from June 2012 to January 2015. Therapy was then interrupted due to persistent stable disease. In November 2016, the disease progressed. The extensive bone marrow infiltration of the tumor precluded a peptide receptor radionuclide therapy (PRRT). In December 2016, the patient was re-exposed to capecitabine and temozolomide until the progression of the liver metastases in February 2017. Consequently, therapy was switched to everolimus treatment. Evidence of pulmonary and hepatic progressive disease appeared in January 2018 leading to discontinuation of everolimus. The ethics committee was consulted before the patient was treated on a single-case basis. The committee granted approval, and the patient gave his informed consent prior to the intervention. In March 2018, a re-biopsy of the liver showed a progressive NET (G3, Ki-67 = 18%). A sample was sent to Ksilink (Strasbourg, France) for personalized functional profiling (PFP), i.e. drug screening of tumor-derived spheroids, and identification of potential hit drugs.

**Figure 1.**
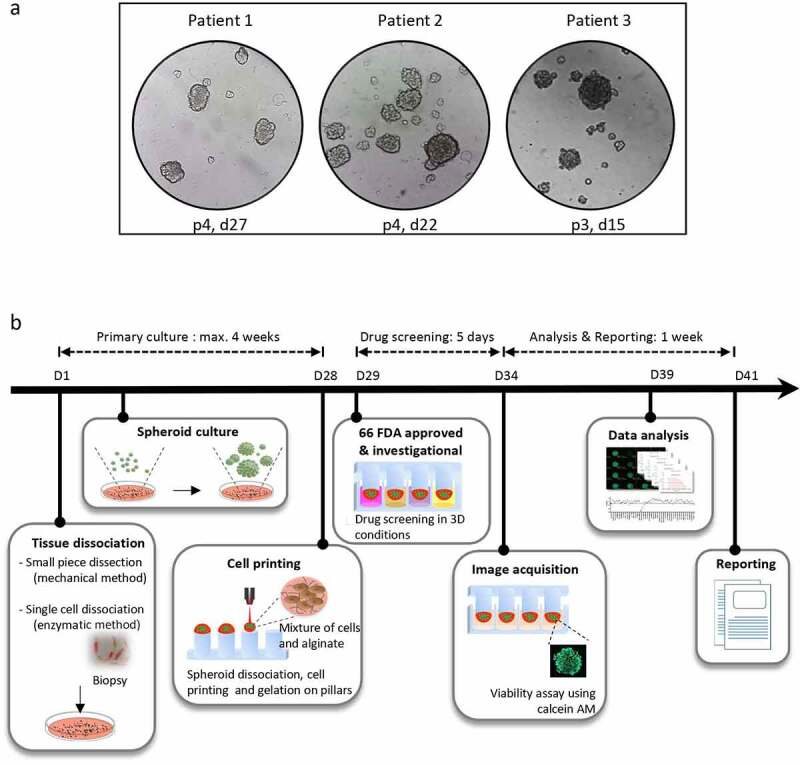
**Representation of the micropillar-based drug screening workflow for personalized functional profiling**. (a) Representative pictures of tumor spheroids. The tumor specimens were obtained from patients 1, 2 and 3 (see Table 1). The images show the spheroids on the indicated passages (p) and days (d) after plating of the original tumor material in ultra-low attachment dishes. The pictures were taken one to 2 days after cell passaging. (b) The tumor biopsy is mechanically and enzymatically dissociated into a single-cell suspension and put in culture for spheroid formation. After maximum 4 weeks, the spheroids are dissociated into single cells and small cell clusters which are dispensed together with the alginate matrix onto a 384-pillar plate using an ASFA Spotter ST (Medical and Bio Decision , South Korea). The pillar plate is then “stamped” with the 384-well plate containing the spheroid growth medium. After one day in culture, the cells are challenged by a panel of 66 FDA-approved or investigational drugs in a fourfold and seven-point serial dilution series from 30 μM to 7.3 nM in duplicates. Cell viability is assessed by calcein AM live cell staining after a 5 day-incubation with the drugs. The IC_50_ and DRC are generated and the AUC is calculated. The patient’s data are then compared to data from other patients to determine the AUC z-score. The treatment is selected as a hit drug for the patient if z-score < −1. The drug screening results are reported to the medical staff approximately 6 weeks after tissue sampling. In panel a, tumor spheroids from 3 patients at different passages and days after cell plating, showing their good propagation in culture. In panel b, steps of a micropillar-based personalized functional profiling, including tissue dissociation, spheroid culture, cell printing in alginate drops, drug screening, image acquisition of calcein AM green fluorescence in live cells, data analysis and report generation.

The collected tumor sample consisted of 4 core-needle biopsies corresponding to 125.5 mg in total (a minimum of 2 needle biopsies is commonly required for spheroid generation). Briefly, the tumor biopsy was mechanically and enzymatically dissociated as follows: The tumor was washed with cold DMEM/F12 supplemented with fetal bovine serum and antibiotics and minced into 1–3 mm^3^ fragments using sterile forceps and a scalpel. Tumor fragments were washed again then digested in DMEM/F12 containing collagenase. The cells were seeded in complete StemPro^TM^ hESC SFM medium (Gibco) in ultra-low attachment dishes, at 37°C in an atmosphere of 5% CO_2_. The cells were regularly inspected under the microscope to check for spheroid formation. The spheroids were passaged every few days using a mild enzymatic dissociation to avoid the accumulation of dead cells in the center of the spheroid. The spheroid culture was deemed successful if the 3D entities displayed a standard rounded multicellular structure and if they could outgrow within days in culture and propagate after passaging. Figure 1(a) shows bright-field images of patient-derived spheroids at variable passages and days after plating, generated from 3 different tumor specimens as described above.

**Figure 2.**
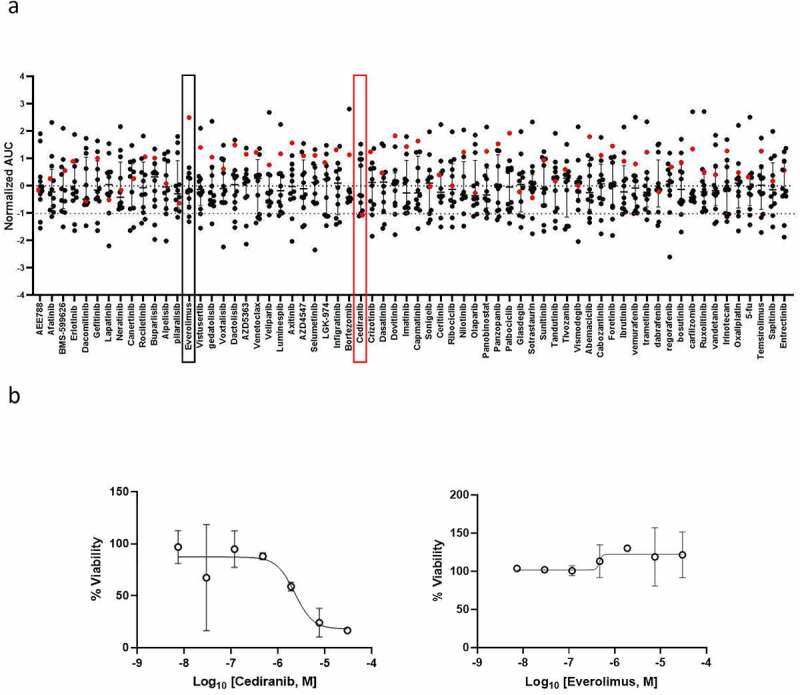
**Drug sensitivity profiles of *ex-vivo* patient-derived spheroids**. Short-term cultured patient-derived spheroids (N = 12 patients) underwent drug sensitivity screening to 66 FDA-approved and investigational drugs. (a) Scatter plot showing normalized AUCs (z-scores from the 12-patient dataset) of the 66 indicated drugs. Each dot represents one patient. The red dot corresponds to the patient described in the present case report and delineates its drug sensitivity profile compared to the other patients. If normalized AUC < −1, the drug is selected as a hit for the patient. In red and black boxes, the results obtained with cediranib and everolimus, respectively. (b) Dose-response curves of *ex-vivo* spheroids from the patient of interest (red dot in a) incubated with increasing concentrations of cediranib (left panel) and everolimus (right panel). The cell viability for each dose was normalized to DMSO-treated cells.

**Figure 3.**
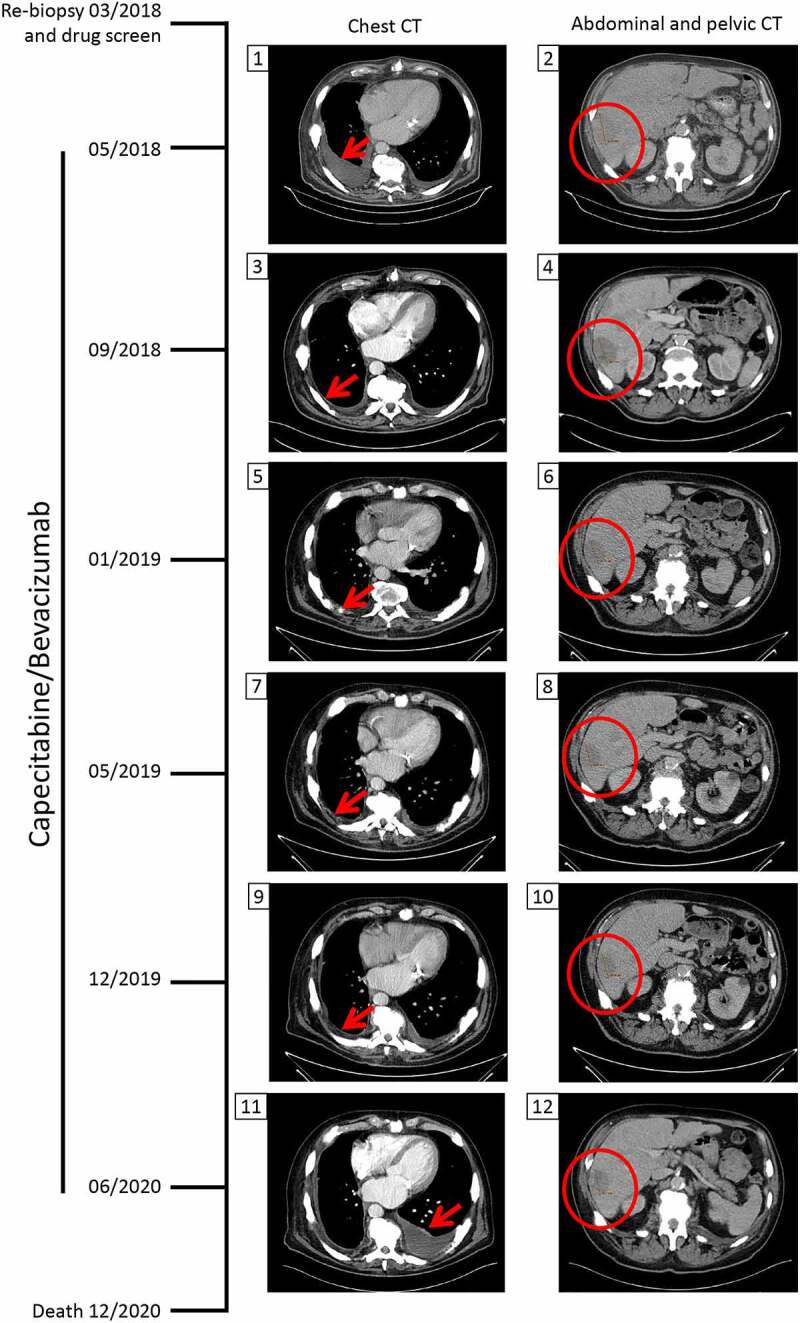
**Chest and abdominal/pelvic computed tomography (CT) scans during capecitabine/bevacizumab treatment**. After evidence of progressive disease, the patient underwent a re-biopsy in March 2018 for *ex-vivo* drug screening. In May 2018, CT scans revealed pleural effusion due to pleural carcinosis and progression of liver metastases. The tumor board decided to treat the patient with capecitabine and bevacizumab based on the personalized functional profiling results and on reported efficacy of this combination in NET. In September 2018, the pleural effusion/pleura carcinosis regressed and a stable disease regarding liver metastasis was noted. With this treatment regimen, the patient maintained a stable disease until its progression in June 2020. The patient died 6 months later. The red arrow shows the appearance, regression and re-appearance of pleural effusion. The red circle marks the liver tumor mass.

PFP was performed on short-term cultured spheroids in order to deliver drug screening results back to the clinician within acceptable timeframes. To do so, the spheroids were dissociated into single cells and small cell clusters and printed with the alginate matrix in hanging drops onto 2 mm-diameter pillars in a 384-pillar plate (with a technical duplicate), using the ASFA Spotter ST (Medical & Bio Decision, Suwon, South Korea) (Figure 1(b)). One day after cell printing, the cells were exposed to a library of 66 FDA-approved and investigational drugs in a fourfold and seven-point serial dilution series for five days. Live cells were stained with calcein AM and the plates were imaged using a high-throughput screening system. The cells were scanned at 4x magnification and their viability was assessed by the area of the calcein AM live cell staining and normalized to the DMSO-treated cells. For each drug, the half-maximal inhibitory concentration (IC_50_) and Dose-Response Curve (DRC) were generated, and the Area under the DRC (AUC) was calculated. To identify personalized drug candidates, we compared the drug sensitivity profiles obtained from the patient tumor spheroids with the pharmacological landscape of 11 other cancer patients’ spheroids. The clinicopathological features of the patients are summarized in Table 1 (patient 11 is the subject of this case report). A drug was considered as a hit of interest for our patient if the AUC z-score was less than −1, indicating the inclusion of the patient’s spheroids in the top 16% most sensitive spheroids to this drug. Based on the drug sensitivity analysis, only cediranib was selected as a hit drug (z-score < −1) (Figure 2(a)). Interestingly, our patient was the most resistant to everolimus (Figure 2(a)), which is in line with the clinical evidence of everolimus resistance manifested in this patient before collecting the tumor sample used for drug screening (Figure 2(a)). The dose-response curves of the patient’s derived spheroids treated with cediranib and everolimus show a dose-dependent toxic effect of cediranib in these cells. In contrast, they were unresponsive to everolimus treatment (Figure 2(b)). Cediranib is a multi-kinase vascular endothelial growth factor receptor (VEGFR) inhibitor that demonstrated promising results in preclinical trials but failed to meet its main goals in several clinical studies.^10–12^ The Next Generation Sequencing assay (TruSight^TM^ Tumor 170-Illumina®) of the liver metastatic sample obtained in March 2018 did not reveal any druggable target, and functional profiling remained the option for improving the disease outcome. Therefore, based on the personalized drug screening results and taking into account the clinical activity and safety profile of the bevacizumab (a VEGF blocker) and capecitabine combination in gastro-intestinal NET in the BETTER trial,^13^ the institutional tumor board recommended treatment with capecitabine and bevacizumab and treatment was initiated in May 2018, after evidence of pleural effusion and progression of liver metastases (Figure 3). Four months later, the pleural effusion and pleura carcinosis regressed and the liver metastasis remained stable (Figure 3). The patient maintained a stable disease during a two-year period under the PFP-guided personalized treatment. In June 2020, 25 months after the start of the capecitabine/bevacizumab treatment, the disease progressed implying acquired resistance to this combination and the patient died 6 months later.

**Table 1.** Clinicopathological features of the patients

Patient’s ID	Diagnosis	Stage	Grade	Anatomic site of the tumor sample used for drug screening
1	Colic adenocarcinoma	IIA	1	Right colon
2	Hepatocellular carcinoma	N.A.	1	N.A.
3	Colic adenocarcinoma	IIIB	2	Right colon
4	Colic adenocarcinoma	IIIB	1	Left colon
5	Colic adenocarcinoma	IIA	1	Right colon
6	Colic adenocarcinoma	IIIB	1	Rectal-sigmoid
7	Colorectal liver metastases	IV	N.A.	Right liver (metastasis)
8	Cholangiocellular carcinoma	IV	N.A.	Liver (metastasis)
9	Neuroendocrine tumor of the pancreas	IV	3	Liver (metastasis)
10	Cholangiocellular carcinoma	IV	N.A.	Liver (metastasis)
11	Atypical carcinoid of the lung	IV	2	Liver (metastasis)
12	Colorectal cancer	IV	2	Liver (metastasis)

**N.A. = information not available**

Computed tomography scan images of the chest and the abdominopelvic region during the co-treatment with capecitabine and bevacizumab. Two months after re-biopsy and drug screening, chest CT showing pleural effusion. Two months after re-biopsy and drug screening, abdominopelvic CT showing progression of liver metastasis. Four months after the start of the co-treatment, regression of pleural effusion. Fig. 4. Four months after the start of the co-treatment, stable liver tumor mass. Fig. 5. Eight months after the start of the co-treatment, stable regression of pleural effusion. Fig. 6. Eight months after the start of the co-treatment, stable liver tumor mass. Fig. 7. One year after the start of the co-treatment, stable regression of pleural effusion. Fig. 8. One year after the start of the co-treatment, stable liver tumor mass. Fig. 9. Nineteen months after the start of the co-treatment, stable regression of pleural effusion. Fig. 10. Nineteen months after the start of the co-treatment, stable liver tumor mass. Fig. 11. Twenty-five months after the start of the co-treatment, reappearance of pleural effusion, implying disease progression. Fig. 12. Twenty-five months after the start of the co-treatment, stable liver tumor mass. Death of the patient 6 months later.

## Discussion

In cancer, angiogenesis, i.e. the formation of new and abnormal blood vessels, is an important factor in tumor growth and metastasis.^14^ The release of pro-angiogenic factors by cancer cells and tumor microenvironment stimulates the migration and proliferation of endothelial cells and triggers vessel formation.^15^ Apart from angiogenesis, other mechanisms account for tumor vascularization, in particular vessel co-option, a process whereby tumor cells incorporate and use preexisting vessels from the surrounding normal tissue to proliferate and spread, and vascular mimicry which is the acquisition by tumor cells of an endothelial-like phenotype leading to vascular-like structures.^15,16^

Previous studies have shown that NET are highly vascularized, suggesting the possible efficacy of antiangiogenic drugs in this indication.^17,18^ Based on this rationale, several clinical trials have been conducted to evaluate the activity of antiangiogenic drugs in advanced NET.^19–21^ These investigations led to the FDA approval of sunitinib in treating progressive, well-differentiated pancreatic NET for patients with locally advanced or metastatic disease.^22^ Although no antiangiogenic drugs have been granted FDA approval for NET of pulmonary origin yet, several clinical trials have already demonstrated their activity in this indication, as illustrated by the results of studies investigating surufatinib,^20^ axitinib,^23^ pazopanib,^24^ and bevacizumab^25^ in NET. Nevertheless, no general treatment recommendation can be issued at this stage regarding the use of antiangiogenic drugs in NET of pulmonary origin.

This case report exemplifies the importance of a personalized functional profiling (PFP) approach for personalized therapy, especially when no other treatment option is expected to generate a promising clinical response. Indeed, with no druggable targets, as revealed by the genomic sequencing, and with the acquisition of secondary drug resistance to several therapies, the decision of pursuing PFP for drug recommendation, i.e. drug screening of tumor-derived spheroids generated from the patient biopsy, and identification of potential hit drugs permitted the selection of an antiangiogenic drug as a potential therapy and confirmed the clinically observed resistance to previous therapies. Accordingly, the patient benefited from an additional two-year period from a personalized treatment based in part on our observation of a significant activity of this class of drugs in an *ex-vivo* spheroid model from the patient’s tumor.

Evasion of anti-angiogenic therapy after an initial response phase has been reported in several metastatic cancers.^26–28^ In this case report, the patient manifested resistance to the capecitabine/bevacizumab combination after two years of treatment. This acquired resistance suggests the activation of adaptive and compensatory mechanisms, e.g. up-regulation of pro-angiogenic factors other than VEGF (targeted by bevacizumab), vascular co-option and vascular mimicry.^15,16^ The cancer stem cell population may also be involved in the secondary drug resistance and tumor relapse, as previously shown.^29,30^ Unfortunately, the degradation of the patient’s health condition was not anymore compatible with a new tumor sampling procedure and thus a follow-up PFP could not be envisaged.

Among the three-dimensional (3D) models used in preclinical research, patient-derived xenografts (PDX) have significantly contributed to the advancement of precision medicine and are still largely used in biomedical research.^31^ Nevertheless, some limitations of these 3D preclinical models are to be mentioned: The possible contribution to the observed drug response of factors inherent to the animal model itself,^32^ the lengthy procedure (6 to 8 months) required for the generation of these PDX models, which may not be compatible with the rapid progression of the disease^33,34^ and the limited number of protocols that can be tested.^31^ Contrarily to a PDX model, our PFP approach exploiting the predictive potential of *ex-vivo* patient-derived spheroids^35,36^ could provide a treatment recommendation in less than two months, a clinically still acceptable timeframe for treatment decision-making in cancer patients. Importantly, our cell culture conditions support the growth and propagation of stem cells, suggesting that at least a fraction of the cells forming the spheroids is composed of cancer cells with stem cell features that may contribute to tumor relapse in the clinic. In addition, our animal-free approach is easily applicable in a hospital environment. Such a personalized strategy is highly required especially in multi-resistant, poorly studied aggressive tumors, as is the case of this metastatic lung atypical carcinoid.
